# Introducing *Cambridge prisms: Precision medicine*

**DOI:** 10.1017/pcm.2023.7

**Published:** 2023-02-14

**Authors:** Anna F Dominiczak, Sandosh Padmanabhan, Mark Caulfield, Ken Sutherland, Jiguang Wang, Jessica K Jones

**Affiliations:** 1 University of Glasgow, Glasgow, UK; 2 Queen Mary University of London, London, UK; 3 Canon Medical Research Europe Ltd., Edinburgh, Scotland, UK; 4 Shanghai Jiaotong University School of Medicine, Shanghai, China; 5 Cambridge University Press & Assessment, Shaftesbury Road, Cambridge, CB2 8EA

**Keywords:** Precision Medicine, Preventive Medicine, Health Economics, Precision Diagnostics, Digital Health

## Defining precision medicine

Precision Medicine (PM) aims to deliver prevention and treatment tailored to individuals’ molecular characteristics. Effective implementation of PM requires seamless integration of laboratory, healthcare data, and decision support systems. Developing and maintaining such a platform relies on global collaborations between clinicians, scientists, patients, healthcare providers, and industry (pharmaceutical, digital companies and healthcare device manufacturers). Governance frameworks must protect and unite patients and communities (equity, public trust, data protection, and privacy), and that research, development, and innovation (rapid open publication, adoption into healthcare systems) are aligned to industry and policy developments (intellectual property protection, regulation, and cost-effectiveness) so that clinical adoption is rapid.

**Figure 1.**
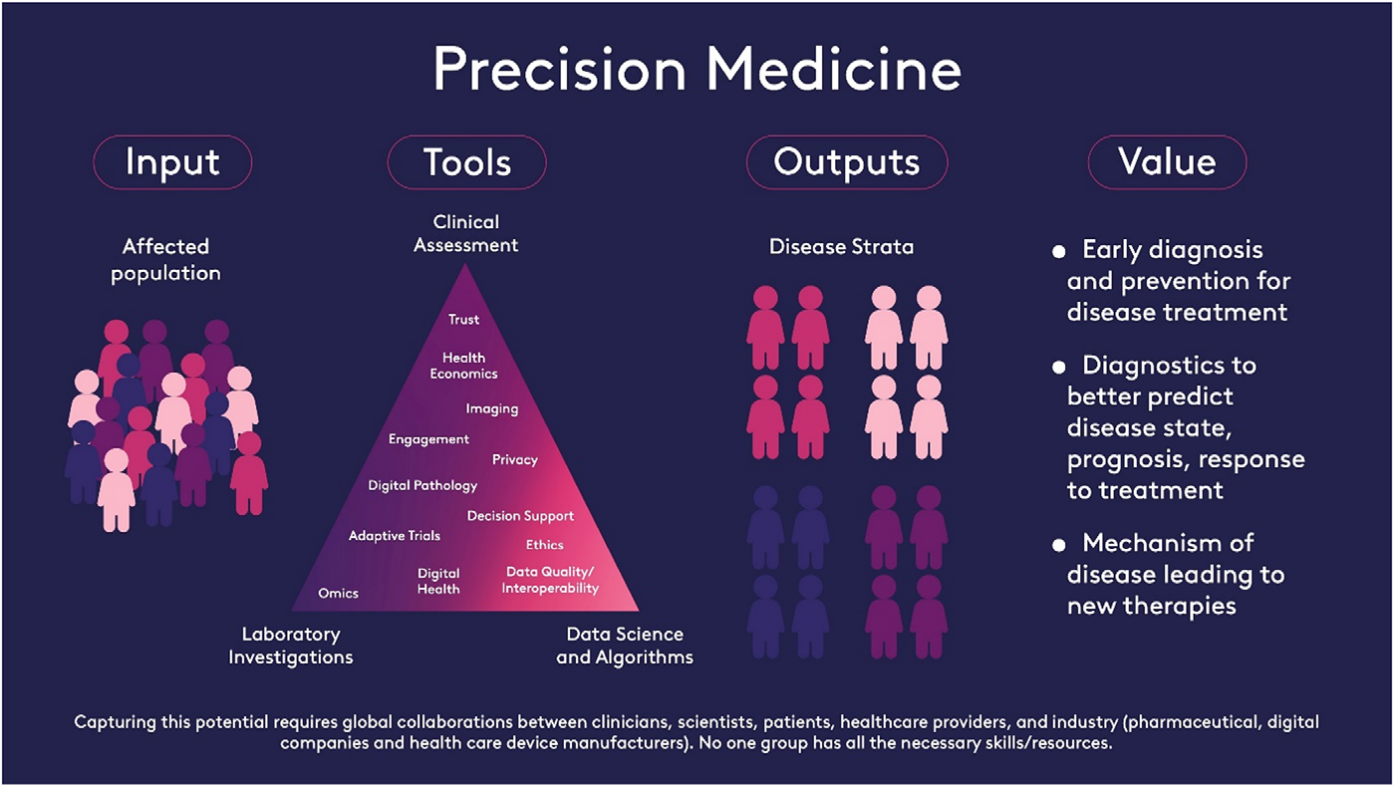
A visualisation of our definition of precision medicine.

- Definition of precision medicine resulting from a collaborative discussion with the Editorial Board of *Cambridge Prisms: Precision Medicine*.
  A schematic covering our the detail behind our definition of PM is given in Figure 1

## Introducing *Cambridge Prisms: Precision Medicine*

Our ambition for *Cambridge Prisms: Precision Medicine* is for it to become a leading international journal, with a focus on interdisciplinary collaborations between academia, healthcare delivery systems, and industry. We would like to publish the best original papers and reviews and to join in the global debates on precision medicine through participation in national and international scientific meetings. We launch the journal with publishing the definition of precision medicine (see above). We thank our Editorial Board members for their contributions to this definition and acknowledge that it is likely to be a subject to dynamic changes as the global community works tirelessly to translate scientific discoveries into healthcare innovations that can be adopted for the benefit of populations world-wide.

“As Regius Professor of Medicine at the University of Glasgow, UK, and Chief Scientist (Health) for the Scottish Government I have been instrumental in strengthening the ‘triple helix’ approach in ensuring successful collaboration between academia, industry, and the National Health Service (NHS) in making precision medicine initiatives affordable and deliverable. I have no doubt precision medicine is the future of medicine.”

Professor Dame Anna Dominiczak, Editor-in-Chief, *Cambridge Prisms: Precision Medicine*

“As former Chief Scientist for the 100,000 Genomes Project, which transformed genomic medicine in the NHS for rare disease, cancer and infection we are clearly in the era of precision healthcare and for that to succeed it must be mainstreamed across the entirety of healthcare and become the province of the many not the few.”

Professor Sir Mark Caulfield, Senior Editor, *Cambridge Prisms: Precision Medicine*

“I work at the intersection of genomics, pharmacogenomics, clinical trials and digital health and witness the technological revolution in biological and clinical data generation and analyses propelling the precision medicine shift in the paradigm of healthcare toward predictive, preventive, participatory, and personalized medicine.”

Professor Sandosh Padmanabhan, Senior Editor, *Cambridge Prisms: Precision Medicine*

“Precision Medicine requires a substantial mind shift within the philosophy and operational delivery of healthcare and even though this transition is large and too complex, we all have a vested interest in improving healthcare outcomes. As an industry leader, I feel privileged to be working closely with a range of clinical and academic experts from many disciplines to find the solutions than can help drive that transition.”

Dr. Ken Sutherland, Senior Editor, *Cambridge Prisms: Precision Medicine*

“My research focuses on the epidemiology and management of hypertension. I am interested in the development of innovative blood pressure measuring techniques and working in the establishment of a web- and app-based blood pressure telemonitoring system for improving awareness and control of hypertension. I am also interested in the investigation of mechanism- or pathogenesis-driven precision treatment for various diseases behind hypertension and ultimately for the prevention and treatment of hypertension-mediated organ damage and complications.”

Dr. Jiguang Wang, Senior Editor, *Cambridge Prisms: Precision Medicine*

## Topic Map

The topic map visualises the journal scope into a journey from fundamental molecular characterisation, **omics,** through to the tools enabling large-scale population genomic sequencing and characterisation, **data science**, with assistance of medical technology, including **precision diagnostics**, to better inform **treatment approaches & therapeutics** and leading to improved prognosis. Meeting the needs of patients through an effective, equitable, and well-governed healthcare system is covered in **health economics**.

## First Content

**Figure 2.**
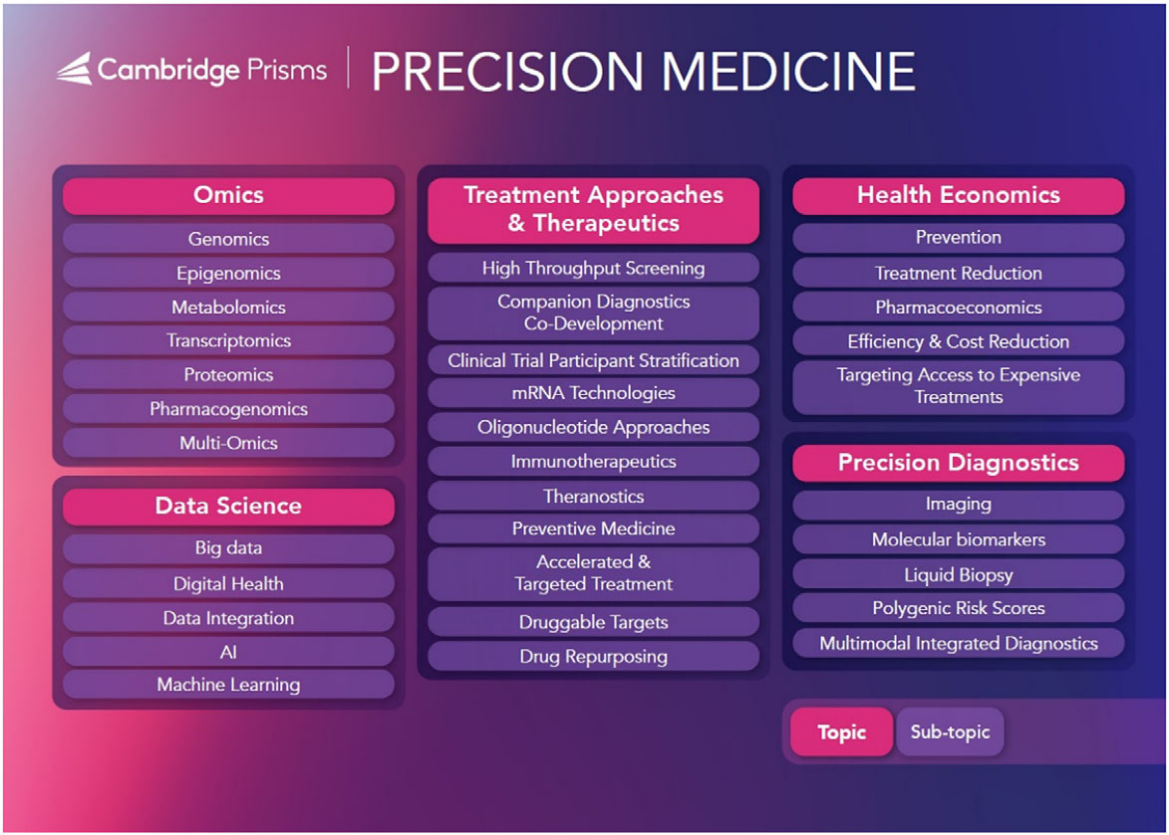
The topic map defining the interdisciplinary scope of *Precision Medicine.*

Prior to launching the journal, the Editors of *Precision Medicine* have invited key researchers, specialists, and practitioners to clearly show our intentions in publishing the latest developments and most prominent discussions in the field from diverse perspectives and expertise with a truly interdisciplinary focus. Topics of the published articles fully map across the journal’s topic map (Figure 2).

The latest developments to be clinically adopted by specialism have been reviewed for the launch of the journal. These articles highlight some of the greatest achievements of precision medicine to date, including within cardiology (Soremekun, 2023), neurodegenerative diseases (Tsoi et al., 2023), oncology (Chamba, 2023; McGough, 2023), mitochondrial medicine (Chinnery, 2022), and also more recent areas of study including blood type susceptibility from severe disease with COVID-19 (Ellinghaus, 2023).

Collaboration across academia, health service delivery systems, and industry, frequently called a triple helix partnership, is essential. It must include pharmaceutical, digital, and artificial intelligence companies as well as heathcare device manufacturers to be effective in delivering clinical utility, laboratory technology, user acceptance, implementation models, and economic value to the field (Jarvis, 2023).

Legal and ethical governance are integral to healthcare frameworks with diversity, equity, and public understanding being key topics under review. A great challenge is in managing the “backdrop of promotional public discourses” that accompany population-level genomic sequencing projects (Horton and Lucassen, 2023), while inequity and lack of inclusion, particularly within clinical trials and polygenic risk scores, remain a great challenge to the field (Geneviève, 2022).

The wider consequences surrounding global adoption of precision medicine must be highlighted and, in some cases, mitigated. For example, in planning for the accompanying environmental footprints of precision medicine large data initiatives (Samuel, 2023).

We look forward to welcoming all contributions on the most pertinent global developments in precision medicine!
